# Supplementary material for: Multidimensional Biomechanics-Based Score to Assess Disease Progression in Duchenne Muscular Dystrophy
Source: Sensors (Basel). 2023 Jan 11;23(2):831. doi: 10.3390/s23020831 (PMC9861677; doi:10.3390/s23020831)
Supplement: Supplementary file 1 [file sensors-23-00831-s001.zip › sensors-2074859-supplementary.pdf]

ANNEX I Clinically relevant parameters

From the before mentioned bibliographic analysis, the previous experience of the biomechanics group, the clinician assessment and the capabilities of the devices used, a set of clinical relevant parameters was defined. All the parameters are classified in five classes depending on the type of variable that represent: spatial-temporal, cardiorespiratory, kinematic, electromyography and plantar pressure. In the following tables (Table S1 – Table S5) all the parameters calculated are listed and defined briefly.

**Spatial-temporal parameters**	
Parameters	Definition
Distance (m)	Distance covered by the patient during the test
Number of steps	Steps walked during the test
Speed (m/s)	Velocity of the patient gait
Cadence (steps/min)	Number of steps per minutes
Stride length (m)	Length in meters of a gait cycle
Stride velocity (m/s)	Velocity in meters / second of a gait cycle
Stance phase (%)	Percentage of time in stance phase, foot- ground contact, on the overall gait cycle.
Swing phase (%)	Percentage of time in swing phase, foot in the air, on the overall gait cycle.
Single Support (%)	Percentage of time in single support, one foot on the ground, on the overall gait cycle.
Double Support (%)	Percentage of time in double support, both feet on the ground, on the overall gait cycle.
Load Response (%)	Percentage of time that takes the load response, absorption of the foot impact, on the overall gait cycle.
Pre – swing (%)	Percentage of time that takes the pre-swing, foot pushed off the ground, on the overall gait cycle.

Table S1: Description of spatial-temporal parameters

**Cardiorespiratory parameters**	
Parameters	Definition
Heart Rate (bpm)	Evolution of the heart rate value throughout the test
Heart Rate range	Difference between the maximum and the minimum HR
R – R intervals	Time intervals between consecutive heart beats
Heart Rate Variability (SDNN)	Variability of RR intervals calculated as the standard deviation of RR intervals

Table S2: Description of heart rate parameters

**Kinematic parameters**	
Parameters	Definition
Hip flexion	Flexion-extension at initial contact (IC)

CONFIDENTIAL

	Flexion-extension at take-off (TO)
	Maximum flexion-extension at swing (Sw)
	Minimum flexion-extension
	Flexion – extension range
Hip adduction	Maximum abduction – adduction
	Minimum abduction – adduction
	Abduction – adduction range
Knee flexion	Flexion – extension at initial contact (IC)
	Flexion – extension maximum at stance (St)
	Flexion – extension minimum at stance (St)
	Flexion – extension maximum at swing (Sw)
	Flexion – extension range
Ankle flexion	Flexion – extension at initial contact (IC)
	Flexion – extension maximum at stance (St)
	Flexion – extension maximum at swing (Sw)
	Flexion – extension minimum at swing (Sw)
	Flexion – extension range

Table S3: Description of kinematic parameters

Electromyography (EMG) parameters	
Parameters	Definition
Maximum voluntary contraction (MVC)	Maximum muscle activation while doing isometric exercises, is related with muscle strength
Muscle Load	1 second EMG mean of each channel. Total muscle load is the sum of the muscle loads of each muscle.
Muscle Distribution	% of activation of each muscle in respect to the total activation
Muscle Balance	% of activation of the left side in comparison to the right side or % of activation of hamstrings in comparison to quadriceps
Co-activation indices	Timing of simultaneous activation of two muscles. Relation between (H/Q, H/G, Q/G).
EMG profiles	Characterized by identifying maximum, minimum, range and values from specific points of the cycle

Table S4: Description of electromyography parameters

Plantar Pressure parameters	
Parameters	Definition
Force Balance (%)	Comparison of the percentage of the total force on right side versus left side
Heel / Toe IC	Detection if initial contact with the ground is with heel or toe
Heel / Toe TO	Detection if take-off the ground is with heel or toe
Centre of Pressure	Trajectory of centre of pressure during gait cycle

Table S5: Description of plantar pressure parameters

CONFIDENTIAL
